# The capability of endophytic fungi for production of hemicellulases and related enzymes

**DOI:** 10.1186/1472-6750-13-94

**Published:** 2013-10-31

**Authors:** Diogo Robl, Priscila da Silva Delabona, Carla Montanari Mergel, Juan Diego Rojas, Patrícia dos Santos Costa, Ida Chapaval Pimentel, Vania Aparecida Vicente, José Geraldo da Cruz Pradella, Gabriel Padilla

**Affiliations:** 1 Institute of Biomedical Sciences, University of São Paulo (USP), Avenida Lineu Prestes 1374 CEP, 05508-900 São Paulo SP, Brazil; 2 Brazilian Bioethanol Science and Technology Laboratory – CTBE, Pólo II de Alta Tecnologia, Rua Giuseppe Maximo Scolfaro 10000, CEP 13083-970 Campinas, SP, Brazil; 3 Departament of Basic Pathology, Federal University of Paraná (UFPR), Caixa Postal 19020, CEP 81531-980 Curitiba, PR, Brazil

**Keywords:** Endophytic fungi, Xylanase, Hemicellulases, Accessory enzymes

## Abstract

**Background:**

There is an imperative necessity for alternative sources of energy able to reduce the world dependence of fossil oil. One of the most successful options is ethanol obtained mainly from sugarcane and corn fermentation. The foremost residue from sugarcane industry is the bagasse, a rich lignocellulosic raw material uses for the production of ethanol second generation (2G). New cellulolytic and hemicellulytic enzymes are needed, in order to optimize the degradation of bagasse and production of ethanol 2G.

**Results:**

The ability to produce hemicellulases and related enzymes, suitable for lignocellulosic biomass deconstruction, was explored using 110 endophytic fungi and 9 fungi isolated from spoiled books in Brazil. Two initial selections were performed, one employing the esculin gel diffusion assay, and the other by culturing on agar plate media with beechwood xylan and liquor from the hydrothermal pretreatment of sugar cane bagasse. A total of 56 isolates were then grown at 29°C on steam-exploded delignified sugar cane bagasse (DEB) plus soybean bran (SB) (3:1), with measurement of the xylanase, pectinase, β-glucosidase, CMCase, and FPase activities. Twelve strains were selected, and their enzyme extracts were assessed using different substrates. Finally, the best six strains were grown under xylan and pectin, and several glycohydrolases activities were also assessed. These strains were identified morphologically and by sequencing the internal transcribed spacer (ITS) regions and the partial β-tubulin gene (BT2). The best six strains were identified as *Aspergillus niger* DR02, *Trichoderma atroviride* DR17 and DR19, *Alternaria* sp. DR45, *Annulohypoxylon stigyum* DR47 and *Talaromyces wortmannii* DR49. These strains produced glycohydrolases with different profiles, and production was highly influenced by the carbon sources in the media.

**Conclusions:**

The selected endophytic fungi *Aspergillus niger* DR02, *Trichoderma atroviride* DR17 and DR19, *Alternaria* sp. DR45, *Annulohypoxylon stigyum* DR47 and *Talaromyces wortmannii* DR49 are excellent producers of hydrolytic enzymes to be used as part of blends to decompose sugarcane biomass at industrial level.

## Background

In nature, lignocellulosic materials are degraded by a consortium of microorganisms that synthesize many hydrolytic enzymes able to loosen and degrade these substrates. Improvement in the efficiency of hydrolysis of lignocellulosic materials has traditionally focused on cellulose, which is the most abundant plant polysaccharide [1]. However, the presence of hemicellulose and lignin can restrict cellulose hydrolysis. The hemicellulases, such as pectinases and xylanases, stimulate cellulose hydrolysis by removal of the non-cellulosic polysaccharides that coat the cellulose fibers [1].

Cellulolytic and hemicellulolytic enzymes have been extensively investigated as tools to achieve viable second-generation ethanol production. The hemicellulases include accessory enzymes, which are a group of enzymes capable of increasing the yield of reducing sugars during enzymatic hydrolysis of lignocellulosic substrates. The definition of the accessory enzymes has evolved over time. Enzymes such as the β-glucosidases were originally classified as accessories, but today are considered essential in enzymatic cocktails, following elucidation of their mechanisms of action during substrate degradation [2-4].

The main accessory enzymes are currently considered to be α-L-arabinofuronosidase, hemicellulolytic esterases, β-mannanases, α-glucoronidases, β-xylosidases, pectinases, and xylanases. Several studies have shown that cellulase enzymes supplementation can improve the enzymatic hydrolysis of lignocellulosic biomass, in terms of speed and hydrolysis yield. An issue is that crude multi-enzyme blends obtained from a single fungus strain are not ideal in biotechnological applications. This is because cellulase activities are not expressed at sufficient levels, or the enzyme complexes are not well balanced in terms of the individual enzymes [3].

For this reason, fungi strains isolated from unusual environments have been sought as alternative sources of hydrolytic enzymes [5,6]. Endophytic fungi are potentially amongst the most interesting microorganisms for screening for the production of industrial biocompounds. These microorganisms are ubiquitous in plants, inhabiting plant tissues without inducing any apparent symptoms in their hosts [7]. The fact that these microorganisms are present within plant tissues could explain their capacity to produce substances that could have useful industrial, agricultural, and medicinal applications [8].

The endophytic fungi that have been reported to be xylanase producers include *Alternaria alternata*[9], *Hymenoscyphus ericae*[10], and *Aspergillus terreus*[11]. De Almeida et al. [12] selected strains from the *Acremonium* endophyte species for hemicellulases and cellulases production. From 14 plant species, Suto et al. [13] isolated 155 strains of fungi that produced xylanases. Harnpicharnchai et al. [14] purified a thermotolerant β-glucosidase from an endophytic *Periconia* sp. Other studies have involved the selection of new isolates using extracellular enzymes as selection parameters for plant growth promotion. Silva et al. [15] investigated fungi isolated from *Annona* spp., while Luz et al. [16] employed isolates from *Passiflora edulis*.

Endophytic fungal strains may therefore constitute a valuable source of biological material that deserves to be studied and explored for the production of cellulolytic and hemicellulolytic enzymes. In this context, the present work concerns the selection of endophytic fungi as producers of hemicellulases and related enzymes with different enzymatic profiles, for use in the deconstruction of lignocellulosic biomass.

## Results

### Agro-industrial waste material composition

The sugar cane hydrothermal pretreatment liquor showed the following composition (g/L): xylo-oligosaccharides (9.98), xylose (4.70), glucose (0.55), arabinose (0.77), cellobiose (0.0), furfural (1.05), hydroxymethylfurfural (0.18), acetic acid (1.47), formic acid (0.23), and total soluble lignin (3.15). Despite the presence of inhibitors, this liquor demonstrated to be a potential carbon source for the screening of enzyme producers and the production of hemicellulases. The DEB was composed of 77.89% cellulose, 7.09% hemicellulose, and 16.22% lignin. The SB consisted of 34% cellulose, 18.13% hemicellulose, 9.78% lignin, and 43.22% protein. The media prepared using these waste materials were therefore able to provide a suitable ratio of cellulose and hemicellulose for the synthesis of glycohydrolases, as well as a good source of nitrogen.

### Plate screening

**Figure 1:**
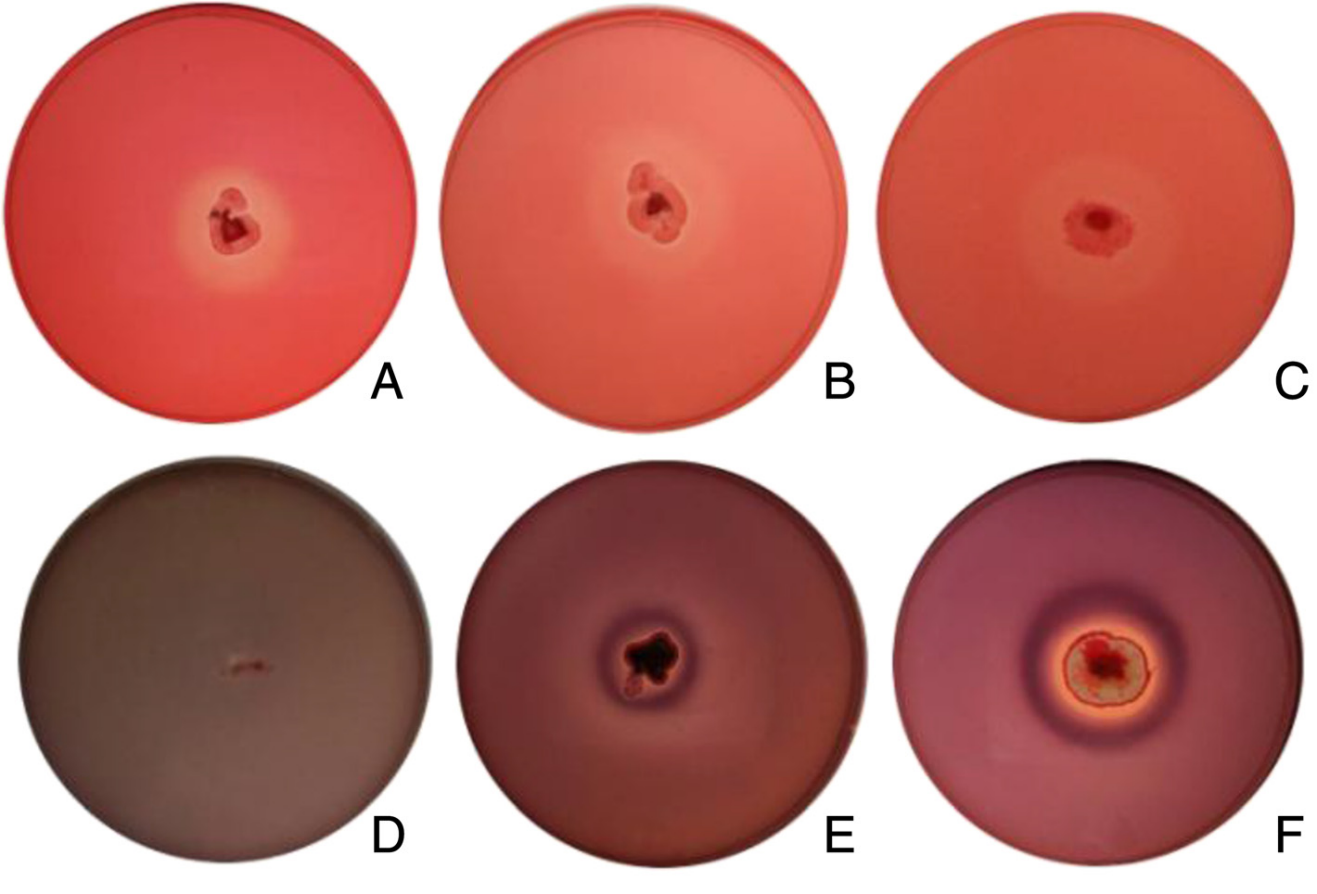
**Hydrolysis results following staining with Congo Red, using xylan agar (A, B, and C) and liquor agar (D, E, and F).** The organisms used were *Penicillium* sp. DR65 **(A,D)**, *Aspergillus* sp. DR06 **(B,E)**, and *Fusarium* sp. DR15 **(C,F)**.

A total of 120 fungal strains were bioprospected and used for calculation of hydrolysis rates (Additional file 1: Table S1). The media containing liquor were stained with Congo Red, revealing the yellow hydrolysis halos (Figure 1). A total of 73 strains were unable to grow on the medium, while only 35 were able to both grow and produce halos. On the other hand, in the case of the medium with xylan, only two strains, one *Aspergillus* sp. and one *Diaphorte* sp. were unable to grow, while 102 strains grew and produced halos. It was therefore demonstrated that the xylose/xylo-oligomers liquor produced by a simple pretreatment was able to sustain the growth of a significant number of the fungi tested.

Selection of β-glucosidase producers employed the EGDA to determine β-glucosidase in the fungal culture extracts, with positive extracts forming dark-colored halos. Of the 119 extracts tested, 63 produced measurable halos, 27 showed dark precipitates although measurement was not possible, and 40 strains were negative for β-glucosidase production. The plate screening and EGDA results were used to select 56 strains for a second screening employing shake flask cultivations. Some of these strains were negative in the hemicellulolytic and β-glucosidase tests, and were used as controls to ensure selection consistency.

### Shake flask screening

**Figure 2:**
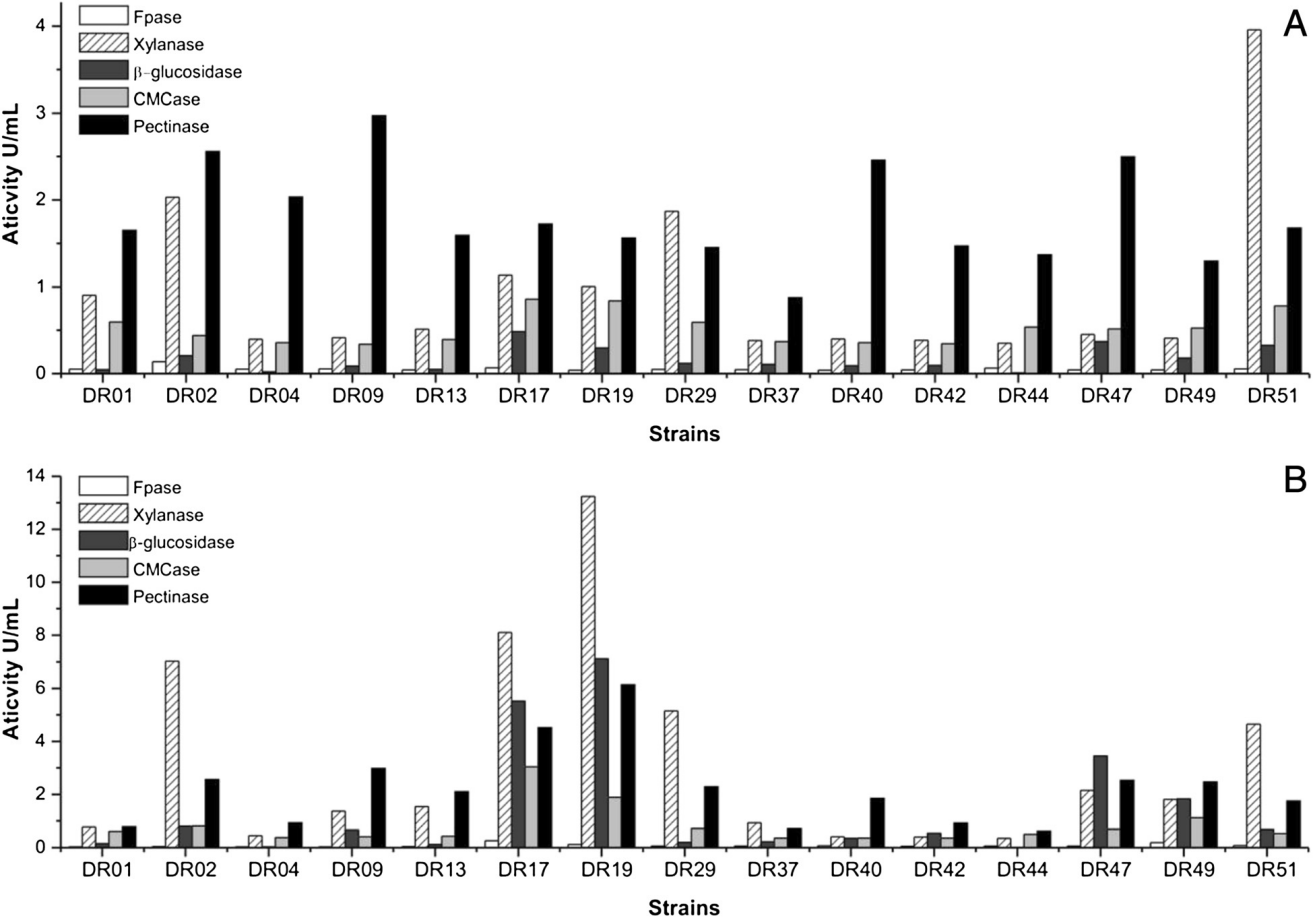
Enzymatic activities of some pre-selected strains, grown in shake flasks with DEB + SB (3:1), after 48 h (A) and 96 h (B).

The strains were grown using DEB + SB (3:1) at 29°C on a rotary shaker at 200 rpm for 96 h. The results obtained for some of the strains are presented in Figure 2. Low β-glucosidase activities were detected up to 48 h of fermentation, while high activity levels were observed at 96 h. This was expected, since several filamentous fungi are known to begin to produce detectable amounts of this enzyme after 72 h of growth [17].

The CMCase and FPase activities were low for all the strains, as expected because selection was performed using materials rich in hemicelluloses. High xylanase production was detected at 48 h for many strains, but the largest peaks occurred at 96 h. Pectinase production showed little variation between 48 and 96 h, although amounts of the enzyme nonetheless increased over the course of the fermentation. Strains morphologically similar to *Aspergillus fumigatus* (DR08, DR03, DR29, and DR31) were excluded due to possible pathogenicity, which could preclude their use in industrial applications.

### Glycohydrolase profile

In order to identify fungi that produced enzyme with different profiles, and hence obtain a more efficient enzymatic extract, 12 strains were selected according to their morphology and enzymatic profiles. A new fermentation with DEB + SB was performed, and samples were taken daily for measurement of xylanase, β-glucosidase, and pectinase activities. The samples that showed the highest glycohydrolase activity were tested using different substrates (Table 1).

**Table 1:** Glycohydrolases activities (U/mL) of twelve selected strains grown using DEB + SB

**Strains**	**DR02**	**DR06**	**DR07**	**DR17**	**DR19**	**DR20**	**DR26**	**DR40**	**DR45**	**DR47**	**DR48**	**DR49**
**Time (h)**	**120**	**120**	**144**	**96**	**72**	**144**	**144**	**120**	**144**	**96**	**144**	**144**
**Birchwood xylan**	4.50	1.38	0.55	10.32	3.22	0.41	0.60	0.38	0.77	0.96	0.50	4.53
**Beechwood xylan**	3.94	2.30	0.95	5.54	3.64	0.63	0.77	0.44	1.11	1.51	1.34	4.03
**Rye arabinoxylan**	2.93	2.19	0.62	4.13	2.00	0.47	0.53	0.38	0.64	1.30	0.44	3.71
**Wheat arabinoxylan**	0.53	0.53	0.36	0.86	0.30	0.36	0.55	0.43	0.45	0.37	0.42	0.27
**Arabinan**	0.46	0.50	0.46	0.48	0.48	0.48	0.47	0.47	0.48	0.49	0.48	0.47
**CMC**	0.27	0.39	0.17	0.26	0.16	0.19	0.18	0.16	0.30	0.22	0.19	0.66
**β-glucan**	1.84	3.63	2.26	4.16	0.53	0.48	2.29	0.47	2.06	2.63	1.35	3.46
**Xyloglucan**	0.52	0.65	0.46	0.73	0.47	0.49	0.48	0.47	0.58	0.69	0.43	0.58
**Lichenan**	1.04	1.81	1.24	2.00	0.66	0.79	1.08	0.37	1.02	1.11	1.14	1.44
**Laminarin**	0.68	0.54	0.76	1.70	0.72	0.59	1.11	0.67	0.72	0.80	1.90	1.44
**Chitosan**	0.63	0.57	0.52	0.53	0.42	0.54	0.68	0.66	0.64	0.61	0.49	0.63
**Glucomannan**	1.02	2.28	0.84	1.83	0.78	0.48	0.95	0.64	1.85	1.64	1.31	1.91
**Galactomannan**	0.75	1.41	0.49	1.79	0.55	0.56	0.53	0.48	1.30	1.24	1.25	1.63
**1,4 β-mannan**	0.65	1.34	0.47	1.22	0.52	0.53	0.50	0.46	1.34	1.44	0.94	1.22
**Pectin**	0.63	0.84	0.69	0.44	0.65	0.87	0.86	0.69	0.77	0.69	0.80	1.14
**pNP β-D-xylopyranoside**	0.13	0.00	0.00	0.01	0.00	0.00	0.00	0.00	0.02	0.01	0.01	0.10
**pNP β-D-mannopyranoside**	0.02	0.00	0.00	0.00	0.00	0.00	0.00	0.00	0.00	0.00	0.00	0.00
**pNP β-D-cellobioside**	0.24	0.03	0.47	0.46	0.15	0.12	0.03	0.18	0.41	0.37	0.35	0.18
**pNP α-L-arabinofuranoside**	0.15	0.02	0.01	0.01	0.00	0.00	0.01	0.02	0.07	0.06	0.02	0.10
**pNP β-D-glucopyranoside**	1.16	0.11	2.85	5.75	0.62	1.19	0.23	0.82	3.44	2.52	1.33	0.68

The strains DR17 and DR19 (*Trichoderma* sp.), and DR02 (*Aspergillus* sp.) presented the highest xylanolytic activities for birchwood xylan, beechwood xylan, and rye arabinoxylan. Despite the fact that the strains DR17 and DR19 belong to the same genus, and have similar morphologies, they presented different enzymatic profiles (Table 1). Selection was made of six strains (DR02, DR17, DR19, DR40, DR47, and DR49) that showed enzymatic activities for a wider range of substrates, were morphologically different, and presented distinct enzymatic profiles. These strains were cultured in shake flasks containing xylan and pectin as inducer carbon sources. Samples were taken daily for measurements of xylanase, β-glucosidase, and pectinase activities. The fungal extracts that showed highest glycohydrolase activities were tested using different substrates (Table 2).

**Table 2:** Glycohydrolases activities (U/mL) of six selected strains grown on pectin and xylan

**Strain**	***A. niger*** **DR02**		***T. atroviride*** **DR17**		***T. atroviride*** **DR19**		***A. stygium*** **DR40**		***Alternaria*** **sp. DR47**		***T. wortmannii*** **DR49**	
**Carbon source**	**Pectin**	**Xylan**	**Pectin**	**Xylan**	**Pectin**	**Xylan**	**Pectin**	**Xylan**	**Pectin**	**Xylan**	**Pectin**	**Xylan**
**Time (h)**	**144**	**144**	**120**	**120**	**96**	**144**	**120**	**144**	**120**	**120**	**120**	**120**
**Birchwood xylan**	0.72	21.34	0.00	2.22	0.59	2.99	1.39	4.68	1.12	1.32	0.74	4.85
**Beechwood xylan**	1.56	15.04	0.60	2.72	0.49	2.88	1.57	6.87	0.00	2.59	1.44	6.00
**Rye arabinoxylan**	1.41	11.15	0.00	2.73	0.61	2.76	1.75	5.98	0.46	1.57	1.12	4.07
**Wheat arabinoxylan**	0.86	3.88	0.80	0.87	0.77	0.87	0.41	0.59	0.15	0.34	0.55	0.37
**Arabinan**	0.52	1.46	0.77	0.48	0.43	0.49	0.55	0.85	0.89	1.11	1.04	0.95
**CMC**	1.32	1.24	0.57	0.56	1.25	0.49	0.36	0.56	0.92	1.28	1.42	4.57
**β-glucan**	2.51	14.03	0.89	1.03	0.69	0.89	0.71	5.67	0.52	1.49	4.16	1.89
**Xyloglucan**	0.47	1.63	0.31	0.47	0.26	0.48	0.49	2.42	0.76	1.38	0.94	0.94
**Lichenan**	1.22	4.66	0.55	0.61	0.74	0.71	0.74	2.37	0.27	0.87	2.14	1.43
**Laminarin**	1.79	1.50	1.72	1.92	1.32	1.78	1.79	4.28	0.66	0.60	3.78	3.20
**Chitosan**	0.60	1.86	0.76	0.63	0.96	0.57	0.88	0.58	0.00	0.00	1.39	1.09
**Glucomannan**	1.34	1.90	0.96	0.72	0.53	0.63	0.74	1.23	0.56	0.79	1.41	1.00
**Galactomannan**	1.11	1.45	0.49	0.49	0.68	0.45	0.50	0.99	0.61	0.23	1.20	0.95
**1,4 β-mannan**	0.87	1.78	0.56	0.59	0.59	0.49	0.54	0.90	0.81	0.54	1.31	1.09
**Pectin**	0.58	0.55	5.09	0.71	4.24	0.49	3.92	1.55	7.72	1.31	1.81	0.72
**pNP β-D-xylopyranoside**	0.16	0.00	0.00	0.05	0.00	0.02	0.03	0.14	0.01	0.02	0.13	2.85
**pNP β-D-mannopyranoside**	0.00	0.00	0.00	0.00	0.00	0.03	0.00	0.00	0.00	0.01	0.04	0.03
**pNP β-D-cellobioside**	0.58	0.00	0.00	0.02	0.00	0.03	0.06	0.50	0.05	0.27	1.15	1.50
**pNP α-L-arabinofuranoside**	0.33	0.21	0.00	0.01	0.00	0.01	0.02	0.67	0.63	0.24	0.57	0.91
**pNP β-D-glucopyranoside**	3.09	0.22	0.05	0.24	0.22	0.30	0.67	1.48	0.52	1.37	1.86	3.13

The fungi xylanolytic profiles differed among the strains and the carbon sources used. The DR17 strain produced xylanases with the same affinity for birchwood xylan, beechwood xylan, and rye arabinoxylan, when cultivated in the presence of beechwood xylan. However, this was not observed when the same *Trichoderma* sp. was grown using DEB + SB. Some strains showed higher activity for beechwood xylan than for birchwood xylan (DR49 and DR40), and vice versa (DR02). The DR02 strain showed the highest activity for rye arabinoxylan. The DR40 strain only produced xylanase when the fungus was grown in the presence of xylan, in contrast to other strains such as DR19, DR49, and DR17, for which DEB and SB also induced the production of xylanases.

The production of β-glucanases was high for DR02 and DR40 strains when cultivated on xylan, for DR17 when grown on DEB + SB, and for DR49 on pectin. However, when these extracts were tested using xyloglucan, all the activities decreased, indicating less affinity for the hydrolysis of β-glucan with branched xylose residues.

A similar phenomenon occurred in the testing of lichenan, which is a linear glucan with more β-1,3 bonds than β-glucan. This indicates that the β-glucanases present in these extracts had lower lichenanase activity. Furthermore, when the DR40 and DR49 strains were grown on xylan, they showed activity against laminarin, indicating the presence of enzymes able to hydrolyze the β-D-glucosyl (1→6) β-D-glucose bond. For almost all fungi, with the exception of DR02 and DR49, the production of polygalacturonase was only induced in the presence of pectin. The best producers were the strains DR47 (7.72 U/mL) and DR17 (5.09 U/mL).

The production of β-glucosidase showed no consistent induction pattern for the three carbon sources tested. DR17 and DR47 produced more β-glucosidase on DEB + SB, while DR02 produced more on pectin, and DR49 on xylan. None of the fungi showed measurable activities for β-1,4-D-glucosaminidase or α-mannosidase.

When the *Talaromyces* sp. DR49 strain was grown on xylan, it was able to produce multiple accessory proteins such as xylosidase, arabinofuranosidase, cellobiohydrolase II, and β-glucosidase. This strain might therefore be promising for the production of hemicellulases. High CMCase activity was measured when this fungus was cultivated on xylan, but it did not present high activities against β-glucan. However, opposite result was found when this strain was grown on DEB + SB.

The hydrolytic action of the fungal extracts against mannan polymers was low for all the strains. Nevertheless, activities for heteromannans (glucomannan and galactomannan) were higher than for β-1,4-mannan. This could be explained by the presence of β-(1→4)-glucanase activity in the extracts in the case of glucomannan, and the presence of α-1,6-galactosidase in the case of galactomannan.

### Fungal identification

**Figure 3:**
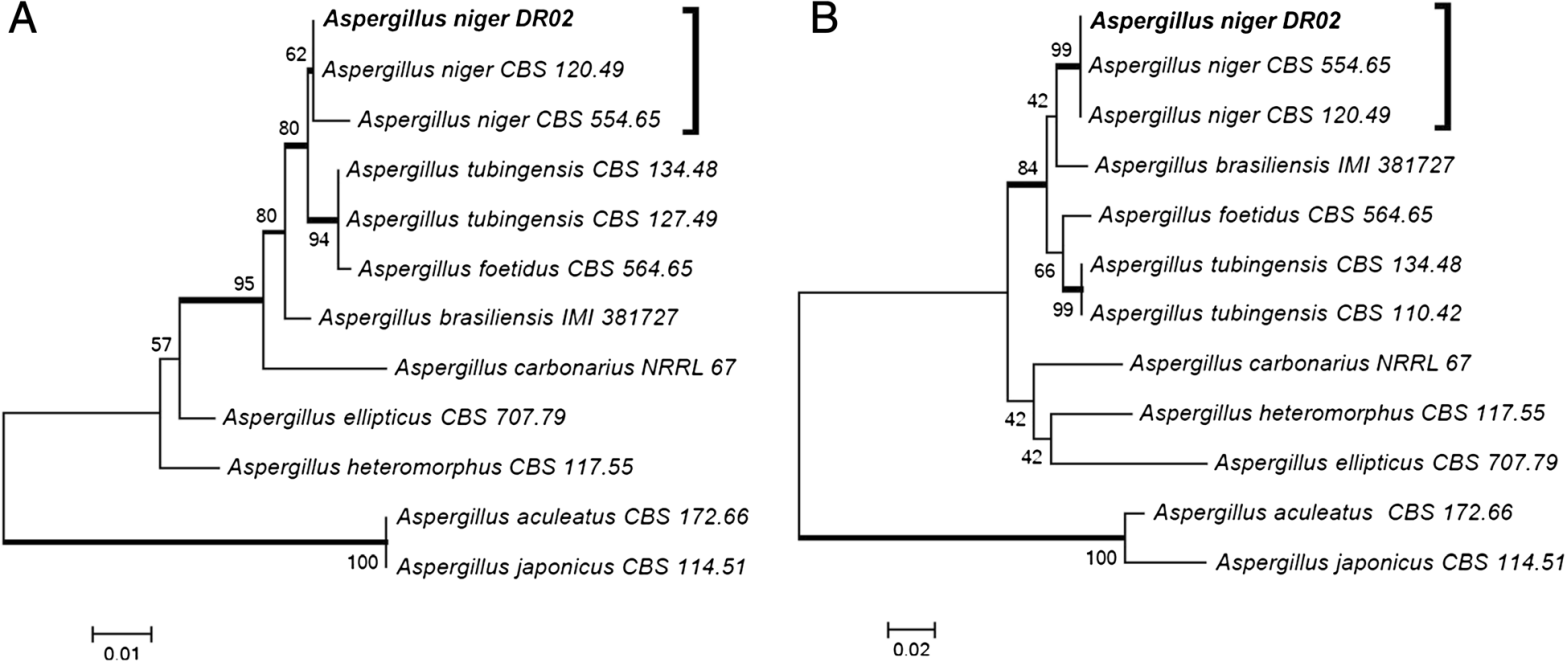
**Phylogenetic tree of *Aspergillus *section *Nigri *based on confidently ITS (A) and partial BT2 (B) sequences constructed with Neighbor-joining implemented in MEGA 4.0.2.** Bootstrap values > 80 from 100 resampled datasets are shown with branches in bold. Strains in bold indicate isolates of this study.

Strain identification was performed using morphological characteristics as well as sequencing of the ITS regions of the ribosomal DNA gene and (in some cases) the partial β-tubulin gene. The best xylanase producer strain, DR02, previously isolated from *Platanus orientalis*, was identified according to morphology (rough dark brown conidia, spherical vesicles and biseriate conidiophores) as *Aspergillus* section *Nigri*. The ITS regions and partial BT2 sequencing were performed and submitted to GenBank (accession number KC311839, KC311845). The phylogenetic trees, built with reference strains of *Aspergillus Nigri* section species, showed that the DR02 isolate clustered with *A. niger* (Figure 3). Higher value of *A. niger* BT2 clustering confirm the ITS result, the strain DR02 belongs to the *Aspergillus niger* species.

**Figure 4:**
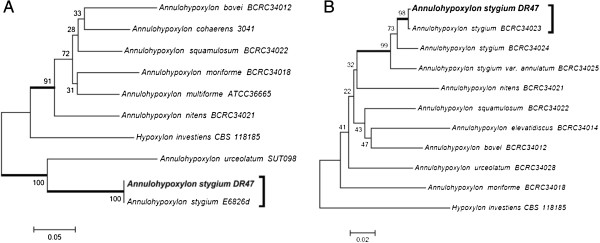
**Phylogenetic tree of *Annulohypoxylon *and related species based on confidently ITS (A) and partial BT2 (B) sequences constructed with Neighbor-joining implemented in MEGA 4.0.2.** Bootstrap values > 80 from 100 resampled datasets are shown with branches in bold. Strains in bold indicate isolates of this study.

The DR47 strain, which is a good pectinase and β-glucosidase producer, did not present reproductive structures under the microculture technique. As the classical methods did not lead to conclusive results, sequencing of the rDNA ITS regions was performed (GenBank accession number KC311843). The blast alignment suggested that the DR47 isolate belonged to the *Annulohypoxylon stygium* species (EU272517, with 99% similarity). A separation of two groups in the ITS tree constructed with *Annulohypoxylon* and related species was found. One group revealed that the DR47 isolated clustered with *A. stygium* and *Annulohypoxylon urceolatum*, but was closer to *A. stygium.* The second group consisted on *Annuhypoxylon* spp. and *Hypoxylon investiens* (Figure 4A)*.* Sánchez-Ballesteros et al. [18] analyzed the ITS1-5.8S-ITS region, and found that *Annulohypoxylon* spp. cluster inter-mingled with species of the genus *Hypoxylon* section *Hypoxylon*. Therefore, sequencing of partial BT2 was also performed (GenBank accession number KC311846) as suggested by Hsieh et al. [19]. The phylogenetic tree was built and the DR47 isolated was clustered with *A. stygium* species, with a high bootstrap value, and was closer to *A. stygium* than to *Annulohypoxylon stygium* var. *annulatum* (Figure 4B). Besides, *H. investiens* was consistently separated from *Annulohypoxylon.*

**Figure 5:**
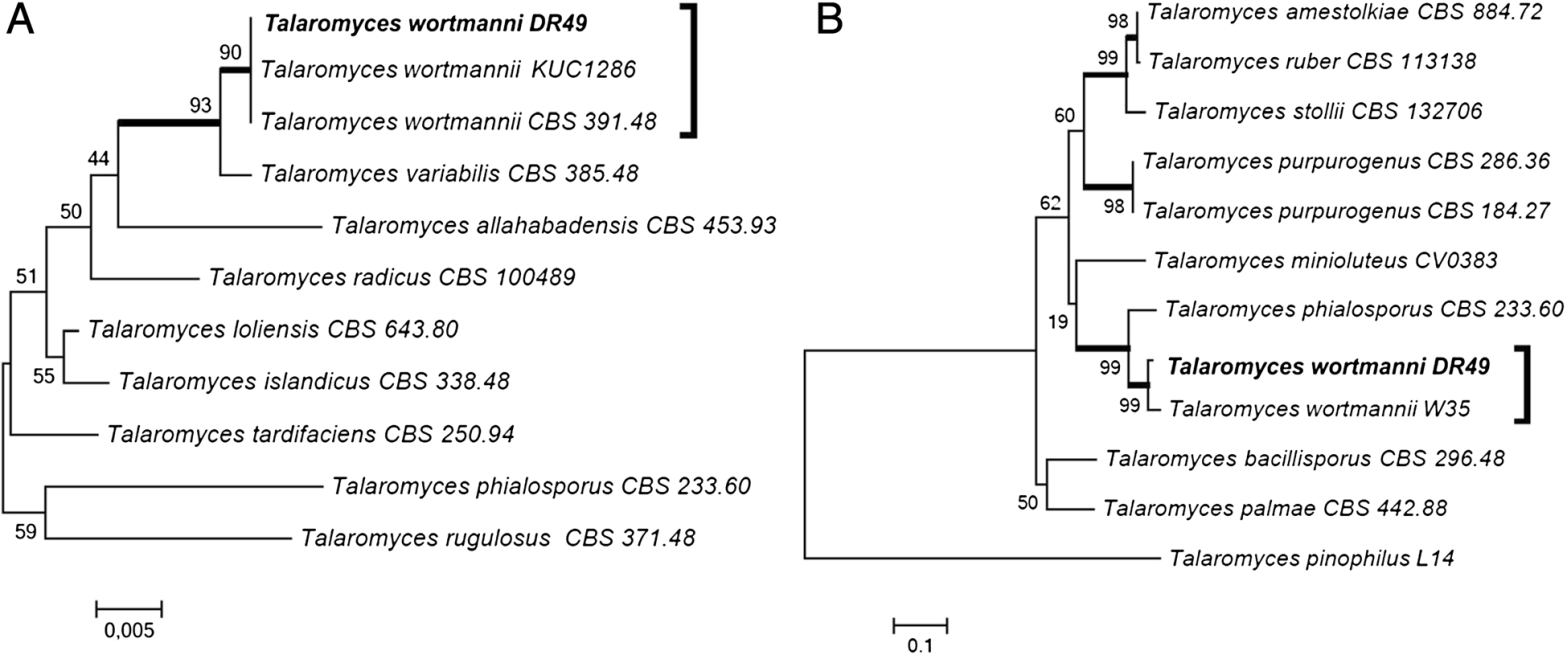
**Phylogenetic tree of *Talaromyces *and close related species based on confidently ITS (A) and partial BT2 (B) sequences constructed with Neighbor-joining implemented in MEGA 4.0.2.** Bootstrap values > 80 from 100 resampled datasets are shown with branches in bold. Strains in bold indicate isolates of this study.

The DR49 strain, previously isolated from spoiled books, was identified as *Talaromyces* sp. The Blast alignment of the ITS regions (GenBank accession number KC311844) and partial BT2 (GenBank accession number KC311847) sequences suggest similarity with to *Talaromyces wortmanni*. The trees based on ITS and BT2 sequencing built with close related *Talaromyces* spp. corroborated with the blast aligned. The *Talaromyces* sp. DR49 strain was clustered with *Talaromyces wortmannii* with high bootstrap values in both trees (Figure 5).

**Figure 6:**
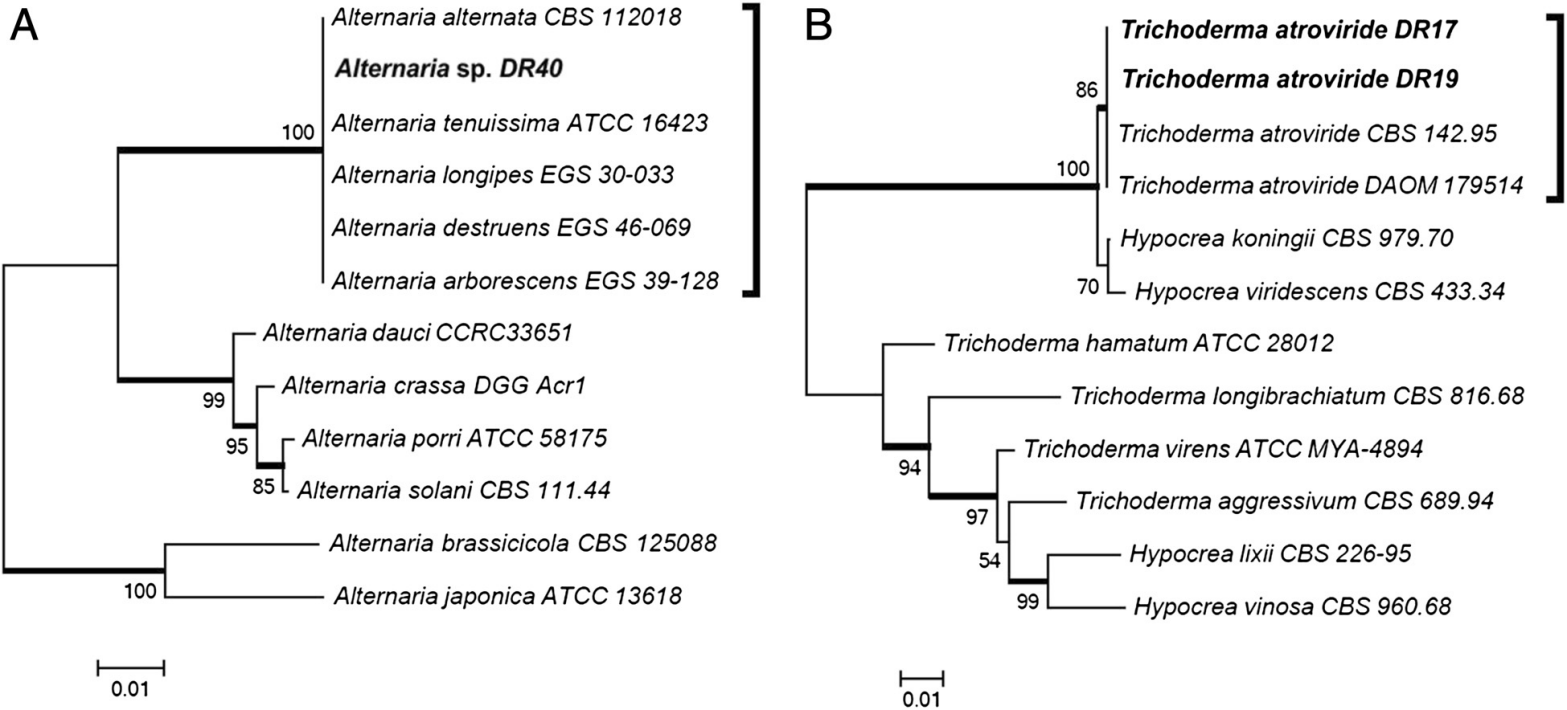
**Phylogenetic tree of *Alternaria *(A) and *Trichoderma *(B) species based on confidently ITS sequences constructed with Neighbor-joining implemented in MEGA 4.0.2.** Bootstrap values > 80 from 100 resampled datasets are shown with branches in bold. Strains in bold indicate isolates of this study.

The DR40 strain, isolated from *E. benthamii*, was previous identified by macro and micro morphology as *Alternaria* sp. The sequencing of rDNA ITS (GenBank accession number KC311842), suggested that the DR40 isolate belonged to the *Alternaria alternata* species (JQ320281, with 100% similarity) while no amplicon of the BT2 gene was obtained for this strain. The tree based on rDNA ITS sequencing built with correlated species showed no resolution among the strains of the *alternata* species group (Figure 6A). Previous work has also found no genetic variation between the small-spored *Alternaria* species in ITS sequences [20,21]. According to Andrew et al. [22], taxonomical differentiation of the small-spored species within the *alternata* group is difficult, not only because there are few distinguishing morphological characteristics, but also because these characteristics are strongly influenced by the environment. Moreover, the same authors could not solve *Alternaria* spp. that belongs to the *alternata* group using a phylogenic multilocus approach.

The DR17 and DR19 strains were also endophytic isolates from *E. benthamii*, and were morphologically identified as *Trichoderma* sp. The ITS1-5,8S-ITS2 sequences for *Trichoderma* sp. DR17 and *Trichoderma* sp. DR19 (GenBank accession numbers (KC311840, KC31184) aligned with the database *Trichoderma atroviride* strain DAOM 179514 with 100% similarity (EU280125). The tree based on rDNA ITS sequencing (Figure 6B) formed two groups, and the DR17 and DR19 isolates were clustered with the *Viride* clade (*T. atroviride, Hypocrea koningii* and *Hypocrea viridescens*), and were closer to the *T. atroviride* species.

## Discussion

High activity, good stability, and low cost are key requirements of enzymes employed for large-scale hydrolysis of lignocellulosic biomass into sugar. Agro-industrial wastes can be useful materials for enzyme development, improvement, and production. The liquor derived from sugar cane bagasse hydrothermal pretreatment is a low cost feedstock [23] rich in xylose and xylo-oligosaccharides which are capable of inducing the expression of xylanases and accessory proteins in fungi such as *A. niger*[24]. Other materials, such as steam-exploded delignified bagasse and soybean bran, have also been used as inexpensive culture media to achieve high xylanase, cellulase, and β-glucosidase activities employing *Trichoderma harzianum* P49P11 [25].

The full hydrolysis of lignocellulosic biomass requires several types of glycohydrolases that enable the release of saccharides and other compounds from the recalcitrant substrate. However, plant species are highly diverse in terms of cell wall structure and composition, which increases the attraction of formulating specific biomass-degrading enzymatic cocktails. The sugar cane cell wall polysaccharide is mainly composed of xyloglucan and arabinoxylan, closely associated with cellulose, as well as pectin, β-glucan and less branched xylan strongly bound to cellulose [26].

Several studies have shown that supplementing cellulases with other enzymes can assist in the enzymatic hydrolysis of lignocellulosic biomass. Xylanases and β-xylosidases improved the hydrolysis yield when combined with cellulases and β-glucosidades [27-29]. The addition of pectinase to Celluclast 1.5 L increased the hydrolysis of pretreated corn stover [1]. Supplementation of cellulolytic cocktails with α-L-arabinofuranosidase and xylanase also showed a synergistic effect in the hydrolysis of wheat straw [30].

The production of glycohydrolases is closely related to the nature of the carbon source, since fungal metabolism is greatly influenced by the composition of the medium (which also hampers the screening of strains). Each strain has a distinct metabolic profile, while the enzymatic profile is also distinct and depends on the medium and the cultivation time (Table 1).

Physiological variations are the result of the adaptation and evolution of fungi, considering their hosts, original habitats, and other factors. The strain *A. stigyum* DR47 belongs to the Xylariaceae family, members of which are frequently encountered as endophytes and saprophytes [31]. Gazis & Chaverri [32] isolated several endophytic Xylariaceae strains and one strain of *Annulohypoxylon* sp. from *Hevea brasiliensis*. Wei et al. [33] cultivated an *A. stigyum* strain on Avicel and confirmed the production of β-glucosidase, although only low levels of cellulases were detected.

Most *Alternaria* species are saprophytes commonly found in soil or on decaying plant tissues, and some species are opportunistic plant pathogens [34]. However, endophytic strains of *Alternaria* spp. have been isolated from eucalyptus plants such as *Eucalyptus globulus*[35] and *Eucalyptus citriodora*[36]. Strains of *A. alternata* are able to produce endopolygalactunorase [37] in the presence of pectin, and β-glucosidase in the presence of saccharose [38].

*A. niger* is known worldwide for its ability to produce an extensive range of extracellular glucohydrolases, including xylanases, pectinases, and β-glucosidase [39]. This characteristic is associated with the ability of the fungus to propagate and colonize a variety of environments, principally those rich in decomposing plant materials [40]. The fungus was recently reported to be endophytic in several plant species [41,42]. However, this work is the first report of *A. niger* as an endophytic fungus in *P. orientalis.*

There have been no previous reports in Brazil concerning *T. wortmannii* isolated from decaying materials. Jang et al. [43] first described β-xylosidase activity in a *T. wortmannii* strain previously isolated from Japanese red pine and larch woods in Korea [44]. Jang et al. [43] obtained a β-xylosidase production of 3.82 U/mL for cultivation on xylan, in good agreement with the β-xylosidase activity (2.85 U/mL) found in the present work for the DR49 strain grown on xylan.

*Trichoderma* spp. are present in soil as saprophytes, and have also been found as endophytic organisms [45]. Many species from this genus are good cellulase and xylanase producers, such as *T. harzianum*[25] and *T. reesei*[46-48]. *T. atroviride* strains are good producers of these glycohydrolases, and can produce high amounts of β-glucosidase [4].

## Conclusions

Microorganisms play an essential role in the degradation of cellulose and hemicellulose standing out the endophytic fungi which are excellent sources of hydrolytic enzymes. Evidently, during the endophytic phase, the use of these enzymes must be related to the mutualistic relationship with the host plant [49]. However, although the association between plants and endophytic fungi is ecologically important, little is known about the physiological characteristics of the interaction.

An important aspect of enzymatic studies involving endophytic fungi is the involvement of these microorganisms in the decomposition of plant material [50,51]. Since the fungi are already present in the senescent plant tissues, they may be able to initiate the decomposition process before it becomes dominated by saprophytic species. This could suggest not only that the production of hydrolytic enzymes by endophytic species might be important for the nutrition of the fungus during the endophytic stage, but also that these enzymes are produced and secreted at the surface of the tissues, where they can compete for the substrate during the saprophytic stage. Kumaresan & Suryanarayanan [52] investigated the ability of endophytic fungi from mangrove leaves of different ages to produce hydrolytic enzymes. It was found that endophytic species occurring at relatively low levels in living leaves were more prevalent after leaf fall, increasing the involvement of these fungi in decomposition of the plant material.

An important consideration is the range of substrates that can be utilized by endophytic microorganisms. Studies have shown that endophytes are capable of metabolizing *in vitro* most substrates found in plants, and produce enzymes including proteases, amylases, phenol oxidases, lipases, laccases, polyphenol oxidases, cellulases, mannanases, xylanases, and pectin lyase [53,54]. The balanced use of microbial enzymes in biomass deconstruction required the understanding of the role played by these glycohydrolases, and also depends on an economic process development. Therefore, biochemical characterization of new reported glycohydrolases producer strains, as well as a bioprocess development of the selected strains in large scale, must be conducted to evaluate the enzyme applicability on the biomass deconstruction, principally on sugar cane bagasse. The present work demonstrated that it is possible to select endophytic fungal strains that can produce glycohydrolases with activities against a wide range of target substrates. This will enable the future formulation of specific enzymatic cocktails for an efficient biomass deconstruction.

## Methods

### Fungal strains

Hemicellulase bioprospection was performed using a fungus culture collection maintained at the Microbiology and Molecular Biology Laboratory of the Federal University of Paraná (LabMicro/UFPR). A total of 119 Brazilian filamentous fungi were selected, previously isolated from *Eucalyptus benthamii*, *Platanus orientalis*, *Glycine max*, *Solanum tuberosum*, *Saccharum officinarum*, and decaying paper. A strain of *Aspergillus niger* ATCC 64973 was used as a positive control in plate assays. The strains were stored on potato dextrose agar (PDA) slants at 4°C.

### Agro-industrial waste materials

The liquor used was derived from the hydrothermal pretreatment of sugar cane bagasse in a reactor (Parr Model 4554, 7.5 L), using 10% (w/w) bagasse, a temperature of 190°C for 10 min, and a 1 h heating gradient. The liquor composition was determined by acid hydrolysis and HPLC [55]. Total soluble lignin was determined by the method described by Gouveia et al. [56]. The sugar cane bagasse was obtained from a local mill (Usina Vale do Rosário, Orlândia, SP, Brazil), and was prepared and characterized by Rocha et al. [57]. The soybean bran (SB) was obtained from Agricola (São Carlos, Brazil) and was characterized by Rodriguez-Zuniga et al. [58].

### Hemicellulolytic plate assay

The selection of hemicellulolytic strains was performed by cultivation on solid medium as described by Kasana et al. [59] containing 0.2% beechwood xylan (Sigma) or aqueous liquor diluted in deionized water at a volume ratio of 25%. The strains were first grown on malt extract agar (MEA) for 5 days at 29°C, and then inoculated onto the test media and incubated for 72 h at 29°C. The pH was adjusted to 5.0, and 0.1% Triton X-100 (Merck) was added as a colony growth limiter. The hydrolysis halos were revealed by application of Congo Red (1%) for 15 min, followed by washing with 1 M NaCl for 10 min [59]. The hydrolysis rates were calculated by dividing the diameters of the hydrolysis halos by the diameters of the colony halos.

### β-glucosidase plate assay

The strains were grown for 5 days in liquid medium [29] with carboxymethylcellulose (CMC, 1%) as sole carbon source, in 10 mL tubes (pH 5.0, 200 rpm, 29°C). The biomass was separated by centrifugation, and the extract was subjected to an esculin gel diffusion assay (EGDA), as described by Saqib & Whitney [60], for 5 h at 37°C. The plate was then placed on ice, and measurement was made of the dark brown zone formed by the action of β-glucosidase on esculin.

### Shake flask cultures

The composition of the main culture medium was adapted from Mandels & Weber [61], using 10 g/L of pretreated delignified sugar cane bagasse (DEB) plus SB, at a 3:1 ratio [25]. The 56 previously selected fungal strains were grown on PDA for 3 days at 29°C, after which one 0.5 cm diameter disc was removed from each colony edge, transferred to an Erlenmeyer flask containing 20 mL of medium, and incubated for 144 h at 29°C and 200 rpm. The best six strains were selected for growth using the same medium described above, but with the carbon source changed to citrus pectin or beechwood xylan. Samples were removed for determination of enzyme activities and protein contents, as described below.

### Enzymatic assays

Measurement of enzymatic activities (in International Units, IU) was performed using different substrates in order to determine global and single activities. Filter paper activity (FPase) was determined as described by Xiao et al. [62]. All the polysaccharides were purchased from Sigma Aldrich or Megazyme, and were assayed at 0.5% in a 10 min reaction. The polysaccharides used were: Beechwood xylan; Birchwood xylan; Rye arabinoxylan; Wheat arabinoxylan; Sugar beet arabinan; CMC; Barley β-glucan; Tamarind xyloglucan; Icelandic moss lichenan; Laminarin from *Laminaria digitata*; Chitosan from shrimp shells; Konjac glucomannan; Carob galactomannan; 1,4 β-mannan and citrus pectin. CMC was assayed in a 30 min reaction. The enzymatic activity was determined from the amount of reducing sugars released from the different polysaccharide substrates, using the DNS method [63] with glucose as standard. The activities of β-glucosidase, β-xylosidase, β-mannosidase, α-L-arabinofuranosidase, and cellobiohydrolase II were measured using the respective p-nitrophenol residues (pNP) (Sigma-Aldrich, USA). The assays employed 10 μL of diluted centrifugation supernatant and 90 μL of the respective pNP (0.5 mM, diluted in citrate buffer), and the mixtures were incubated for 10 min at 50°C. The reactions were stopped by adding 100 μL of 1 M Na_2_CO_3_, and the absorbance was measured at 400 nm using a Tecan Infinite® 200 instrument (Männedorf, Switzerland). All the assays utilized an epMotion® 5075 automated pipetting system (Eppendorf) and were performed at pH 5,0 with 50 mM citrate buffer. One unit of glycohydrolases activity corresponds to 1 μmol of glucose or pNP released per minute.

### Morphological identification

Initial fungus identification was performed using macro and micro morphological characteristics [64-66]. The analysis of fungal reproductive structures by optical microscopy was carried out as described by Kern & Blevins [67].

### DNA extraction

An approximately 1 cm^2^ colony of 5-day-old cultures was transferred to a 2 mL Eppendorf tube containing 300 μL CTAB (cetyltrimethylammonium bromide) buffer (2% (w/v) CTAB, 1.4 M NaCl, 100 mM Tris–HCl, pH 8.0, 20 mM EDTA, and 0.2% (v/v) β-mercaptoethanol) and about 80 mg of a 2:1 (w/w) mixture of silica gel H (Merck) and Celite™ 545 (Macherey Nagel & Co). The cells were disrupted manually with a sterile pestle for about 5 min. Subsequently, 200 μL CTAB buffer was added, and the mixture was vortexed and then incubated for 10 min at 65°C. After the addition of 500 μL chloroform, the solution was mixed and centrifuged for 5 min at 20,500 × *g*. The supernatant was transferred to a new tube, together with 2 volumes of ice-cold 96% ethanol. The DNA was allowed to precipitate for 30 min at -20°C, after which centrifugation was performed for 5 min at 20,500 × *g*. After washing with cold 70% ethanol and drying at room temperature, the pellet was resuspended in 97.5 μL TE buffer together with 2.5 μL RNAse (20 U/mL), and incubated for 5 min at 37°C, before storage at -20°C [68].

### DNA amplification and sequencing

The rDNA Internal Transcribed Spacer (ITS) region was amplified using ITS5 and ITS4 primers [69]. Partial β-tubulin (BT2) gene was amplified using Bt2a and Bt2b primers [70]. The sequencing of β-tubulin gene was performed for some strains to confirm the ITS phylogeny clustering. Amplicons were cleaned with a GFX™ PCR DNA purification kit (GE Healthcare, UK). Sequencing was performed on an ABI 3130 automatic sequencer (Applied Biosystems). The Staden sequence analysis package (v. 1.6.0) was used to edit and align the sequences [71]. Sequence analysis was performed using BLASTn sequence alignment software, run against the NCBI (National Center for Biotechnology Information) database. The phylogenetic trees were constructed with 1000 bootstrap replicates using MEGA v4.0.2 software [72], with application of the neighbor-joining method [73], the Jukes-Cantor distance correction model [74]. The nucleotide sequences used in this study were obtained/submitted to GenBank (Additional file 1: Table S2).

## Abbreviations

DEB: Deglignified sugar cane bagasse; SB: Soybean bran; CMC: Carboxymethylcellulose; FP: Filter paper; ITS: Internal transcribed spacer; BT2: β-tubulin gene; EGDA: Esculin gel diffusion assay; pNP: p-nitrophenol.

## Competing interests

JGP is employed at CTBE; GP at USP, ICP and VAV at UFPR; CMM, PSD, PSC and DR are M.Sc and Ph.D students respectively; JDR posdoctoral at USP.

## Authors’ contributions

GP, JGP, JDR, DR conceived the study and wrote the paper; DR, CMM, PSD, PSC produced the biological, enzymatic data; JDR and DR performed phylogenetic studies; ICP, VAV, JDR and DR isolated, preserved and identified fungal strains. All authors read and approved the manuscript.

## Supplementary Material

Additional file 1: Table S1 Hydrolysis rate of the bioprospected fungal strains. **Table S2.** Nucleotide sequences of fungal strains submitted to GenBank. Click here for file

## References

[B1] Berlin A Maximenko V Gilkes N Saddler J Optimization of enzyme complexes for lignocellulose hydrolysis Biotechnol Bioeng 2007 97 287 296 10.1002/bit.21238 17058283

[B2] Gao D Chundawat SPS Krishnan C BalanV DBE Mixture optimization of six core glycosyl hydrolases for maximizing saccharification of ammonia fiber expansion (AFEX) pretreated corn stover Bioresour Technol 2010 101 2770 2781 10.1016/j.biortech.2009.10.056 19948399

[B3] Gusakov AV Salanovich TN Antonov AI Ustinov BB Okunev ON Burlingame R Emalfarb M Baez M Sinitsyn AP Design of highly efficient cellulase mixtures for enzymatic hydrolysis of cellulose Biotechnol Bioeng 2007 97 1028 1038 10.1002/bit.21329 17221887

[B4] Kovács K Megyeri L Szakacs G Kubicek CP Galbe M Zacchi G *Trichoderma atroviride* mutants with enhanced production of cellulase and β-glucosidase on pretreated willow Enzyme Microb Technol 2008 43 48 55 10.1016/j.enzmictec.2008.02.006

[B5] Maijala P Kango N Szijarto N Viikari L Characterization of hemicellulases from thermophilic fungi Antonie Van Leeuwenhoek 2012 101 905 917 10.1007/s10482-012-9706-2 22371150

[B6] Panno L Bruno M Voyron S Anastasi A Gnavi G Miserere L Varese GC Diversity, ecological role and potential biotechnological applications of marine fungi associated to the seagrass *Posidonia oceanica* N Biotechnol 2013 doi:10.1016/j.nbt.2013.01.010 10.1016/j.nbt.2013.01.010 23410985

[B7] Zabalgogeazcoa I Oleaga A Pérez-Sánchez R Pathogenicity of endophytic entomopathogenic fungi to *ornithodoros erraticus* and *ornithodoros moubata* (acari: argasidae) Vet Parasitol 2008 158 336 343 10.1016/j.vetpar.2008.09.019 18976863

[B8] Huang Z Cai X Shao C She Z Xia X Chen Y Yang J Zhou S Lin Y Chemistry and weak antimicrobial activities of phomopsins produced by mangrove endophytic fungus Phomopsis sp. ZSU-H76 Phytochemistry 2008 69 1604 1608 10.1016/j.phytochem.2008.02.002 18343465

[B9] Wipusaree N Sihanonth P Piapukiew J Sangvanich P Karnchanatat A Purification and characterization of a xylanase from the endophytic fungus *alternaria alternata* isolated from the Thai medicinal plant, *croton oblongifolius* roxb Afr J Microbiol Res 2011 5 5697 5712

[B10] Burke RM Cairney JWG Purification and characterization of a β-1,4-endoxylanase from the ericoid mycorrhizal fungus *Hymenoscyphus ericae* New Phytol 1997 35 345 352

[B11] Sorgatto M Guimarães NCA Zanoelo FF Marques MR Peixoto-Nogueira SC Giannesi GG Purification and characterization of an extracellular xylanase produced by the endophytic fungus, *Aspergillus terreus*, grown in submerged fermentation Afr J Biotechnol 2012 11 8076 8084

[B12] de Almeida MN Guimarães VM Bischoff KM Falkoski DL Pereira OL Gonçalves DS de Rezende ST Cellulases and hemicellulases from endophytic acremonium species and its application on sugarcane bagasse hydrolysis Appl Biochem Biotechnol 2012 165 594 610 21573756 10.1007/s12010-011-9278-z

[B13] Suto M Takebayashi M Saito K Tanaka M Yokota A Tomita F Endophytes as producers of xylanase J Biosci Bioeng 2002 93 88 90 16233170

[B14] Harnpicharnchai P Champreda V Sornlake W Eurwilaichitr L A thermotolerant beta-glucosidase isolated from an endophytic fungi, *Periconia* sp., with a possible use for biomass conversion to sugars Protein Expr Purif 2009 67 61 69 10.1016/j.pep.2008.05.022 18602476

[B15] Silva RLO Luz JS Silveira EB Cavalcante UMT Fungos endofíticos em *annona* spp.: isolamento, caracterização enzimática e promoção do crescimento em mudas de pinha (*annona squamosa* L.) Acta Bot Bras 2006 20 649 655 10.1590/S0102-33062006000300015

[B16] Luz JS Silva RLO Silveira EB Cavalcante UMT Atividade enzimática de fungos endofíticos e efeito na promoção do crescimento de mudas de maracujazeiro-amarelo Caatinga 2006 19 128 134

[B17] Castro AM Ferreira MC da Cruz JC Pedro KC Carvalho DF Leite SG Pereira N High-yield endoglucanase production by *trichoderma harzianum* IOC-3844 cultivated in pretreated sugarcane mill byproduct Enzyme Res 2010 2010 854526 21048871 10.4061/2010/854526 PMC2962913

[B18] Sánchez-Ballesteros J González V Salazar O Acero J Portal MA Julián M Rubio V Phylogenetic study of *hypoxylon* and related genera based on ribosomal ITS sequences Mycologia 2000 92 964 977 10.2307/3761591

[B19] Hsieh HM Ju YM Rogers JD Molecular phylogeny of *hypoxylon* and closely related genera Mycologia 2005 97 844 865 10.3852/mycologia.97.4.844 16457354

[B20] Chou HH Wu WS Phylogenetic analysis of internal transcribed spacer regions of the genus *Alternaria*, and the significance of filament-beaked conidia Mycol Res 2002 106 164 169 10.1017/S0953756201005317

[B21] Pryor BM Bigelow DM Molecular characterization of *embellisia* and *nimbya* species and their relationship to *alternaria*, *ulocladium* and *stemphylium* Mycologia 2003 95 1141 1154 10.2307/3761916 21149017

[B22] Andrew M Peever TL Pryor BM An expanded multilocus phylogeny does not resolve morphological species within the small-spored *alternaria* species complex Mycologia 2009 101 95 109 10.3852/08-135 19271672

[B23] Silva VFN Arruda PV Felipe MGA Gonçalves AR Rocha GJM Fermentation of cellulosic hydrolysates obtained by enzymatic saccharification of sugarcane bagasse pretreated by hydrothermal processing J Ind Microbiol Biotechnol 2011 38 809 817 10.1007/s10295-010-0815-5 20740373

[B24] De Vries RP Visser J de Graaff LH CreA modulates the XlnR-induced expression on xylose of *Aspergillus Niger* genes involved in xylan degradation Res Microbiol 1999 150 281 285 10.1016/S0923-2508(99)80053-9 10376490

[B25] Delabona Pda S Farinas CS Lima DJ Pradella JG Experimental mixture design as a tool to enhance glycosyl hydrolases production by a new *Trichoderma harzianum* P49P11 strain cultivated under controlled bioreactor submerged fermentation Bioresour Technol 2013 132 401 405 23265822 10.1016/j.biortech.2012.11.087

[B26] Souza AP Leite DCC Pattathil S Hahn MG Buckridge MS Composition and structure of sugarcane cell wall polysaccharides: implications for seconf-generation bioethanol production Bioenerg Res 2012 doi:10.1007/s12155-012-9268-1

[B27] Gao D Uppugundla N Chundawat SPS Yu X Hermanson S Gowda K Brumm P Mead D Balan V Dale BE Hemicellulases and auxiliary enzymes for improved conversion of lignocellulosic biomass to monosaccharides Biotechnol Biofuels 2011 4 5 10.1186/1754-6834-4-5 21342516 PMC3056733

[B28] Gottschalk LMF Oliveira RA Bom EPS Cellulases, xylanases, β-glucosidase and ferulic acid esterase produced by *Trichoderma* and *Aspergillus* act synergistically in the hydrolysis of sugarcane bagasse Biochem Eng J 2010 51 72 78 10.1016/j.bej.2010.05.003

[B29] Kumar R Wyman CE Effect of xylanase supplementation of cellulase on digestion of corn stover solids prepared by leading pretreatment technologie Bioresour Technol 2009 100 4203 4213 10.1016/j.biortech.2008.11.057 19386492

[B30] Alvira P Negro MJ Ballesteros M Effect of endoxylanase and a-L-arabinofuranosidase supplementation on the enzymatic hydrolysis of steam exploded wheat straw Bioresour Technol 2011 102 4552 4558 10.1016/j.biortech.2010.12.112 21262567

[B31] Stone J Polishook J White-Jr F Foster M, Bills G, Mueller G Endophytic fungi Biodiversity of fungi: inventory and monitoring methods. 1st Edition 2004 New York: Academic Press 241 270

[B32] Gazis R Chaverri P Diversity of fungal endophytes in leaves and stems of wild rubber trees (Hevea brasiliensis) in Peru Fungal Ecol 2010 3 240 254 10.1016/j.funeco.2009.12.001

[B33] Wei DL Chang SC Wei YH Lin YW Chuang CL Jong SC Production of cellulolytic enzymes from the *Xylaria* and *Hypoxylon* species of xylariaceae World J Microbiol Biotechnol 1992 8 141 146 10.1007/BF01195834 24425396

[B34] Thomma BPHJ *Alternaria* spp.: from general saprophyte to specific parasite Mol Plant Pathol 2003 4 225 236 10.1046/j.1364-3703.2003.00173.x 20569383

[B35] Lupo S Tiscornia S Bettucci L Endophytic fungi from flowers, capsules and seeds of *Eucalyptus globules* Rev Iberoam Micol 2001 18 38 41 15482013

[B36] Kharwar RNG Surendra KKA Mishra A A comparative study of endophytic and epiphytic fungal association with leaf of *Eucalyptus citriodora* Hook., and their antimicrobial activity World J Microbiol Biotechnol 2010 26 1941 1948 10.1007/s11274-010-0374-y

[B37] Isshiki A Akimitsu K Nishio K Tsukamoto M Yamamoto H Purification and characterization of an endopolygalacturonase from the rough lemon pathotype of *Alternaria alternata*, the cause of citrus brown spot disease Physiol Mol Plant Pathol 1997 51 155 167 10.1006/pmpp.1997.0106

[B38] Sáenz-de-Santamaría M Guisantes JA Martínez J Enzymatic activities of *Alternaria alternata* allergenic extracts and its major allergen (Alt a 1) Mycoses 2006 49 288 292 10.1111/j.1439-0507.2006.01238.x 16784442

[B39] Ward OP Qin WM Dhanjoon J Ye J Singh A Physiology and biotechnology of *Aspergillus* Adv Appl Microbial 2005 58 1 75 10.1016/S0065-2164(05)58001-8 16543029

[B40] Meijer M Houbraken JAMP Dalhuijsen S Samson RA Vries RP Growth and hydrolase profiles can be used as characteristics to distinguish *Aspergillus Niger* and other black aspergilla Stud Mycol 2011 69 19 30 10.3114/sim.2011.69.02 21892240 PMC3161755

[B41] Ilyas M Kanti A Jamal Y Herdina AA Biodiversity of endophytic fungi associated with *uncaria Gambier* roxb. (Rubiaceae) from west Sumatra Biodiversitas 2009 10 23 28

[B42] Zhao K Ping W Li Q Hao S Zhao L Gao T Zhou D *Aspergillus Niger* var. Taxi, a new species variant of taxol-producing fungus isolated from *taxus cuspidate* in China J Appl Microbiol 2009 107 1202 1207 10.1111/j.1365-2672.2009.04305.x 19486395

[B43] Jang Y Lee J Lee H Lee S Kim G Kim J Screening for xylanase and β-xylosidase production from wood-inhabiting *Penicillium* strains for potential use in biotechnological applications Holzforschung 2012 66 267 271

[B44] Lee J Jang Y Lee H Lee S Kim G Kim J Phylogenetic analysis of major molds inhabiting woods and their discoloration characteristics. Part 2. Genus *penicillium* Holzforschung 2011 65 265 270

[B45] Xia X Lie TK Qian X Zheng Z Huang Y Shen Y Species diversity, distribution, and genetic structure of endophytic and epiphytic Trichoderma associated with banana roots Microb Ecol 2011 61 619 625 10.1007/s00248-010-9770-y 21063870

[B46] Ahamed A Vermette P Culture-based strategies to enhance cellulase enzyme production from *Trichoderma reesei* RUT-C30 in bioreactor culture conditions Biochem Eng J 2008 40 399 407 10.1016/j.bej.2007.11.030

[B47] Ahamed A Vermette P Effect of culture medium composition on *Trichoderma reesei*’s morphology and cellulase production Bioresour Technol 2009 100 5979 5987 10.1016/j.biortech.2009.02.070 19592237

[B48] Olsson L Christensen TMIE Hansen KP Palmqvist EA Influence of the carbon source on production of cellulases, hemicellulases and pectinases by *Trichoderma reesei* Rut C – 30 Enzyme Microb Technol 2003 33 612 619 10.1016/S0141-0229(03)00181-9

[B49] Moy M Li HM Sullivan R White JF Jr Belanger FC Endophytic fungal beta-1,6-glucanase expression in the infected host grass Plant Physiol 2002 130 1298 1308 10.1104/pp.010108 12427996 PMC166650

[B50] Müller MM Valjakka R Suokko A Hantula J Diversity of endophytic fungi of single Norway spruce needles and their role as pioneer decomposers Mol Ecol 2001 10 1801 1810 10.1046/j.1365-294X.2001.01304.x 11472547

[B51] Petrini O Andrews JHSS Fungal endophyte of tree leaves Microbial ecology of leaves 1991 New York: Spring-verlag 179 197

[B52] Kumaresan V Suryanarayanan TS Endophyte assemblages in young, mature and senescent of *Rhizophora apiculata*: evidence for the role of endophytes in mangrove litter degradation Fungal Divers 2002 9 81 91

[B53] Lumyong S Lumyong P McKenzie EH Hyde KD Enzymatic activity of endophytic fungi of six native seedling species from Doi Suthep-Pui National Park, Thailand Can J Microbiol 2002 48 1109 1112 10.1139/w02-112 12619825

[B54] Schulz B Boyle C The endophytic continuum Mycol Res 2005 109 661 686 10.1017/S095375620500273X 16080390

[B55] Sluiter A Hames B Ruiz R Scarlata C Sluiter J Templeton D Determination of sugars, byproducts, and degradation products in liquid fraction process samples Standard biomass analytical procedures Available http://www.nrel.gov/docs/gen/fy08/42623.pdf Accessed 23 Fev 2013

[B56] Gouveia ER Nascimento RT Souto-Maior AM Rocha GJM Validação de metodologia para a caracterização química de bagaço de cana-de-açúcar Quim Nova 2009 32 1500 1503 10.1590/S0100-40422009000600026

[B57] Rocha GJM Gonçalves AR Oliveira BR Olivares EG Rossel CEV Steam explosion pretreatment reproduction and alkaline delignification reactions performed on a pilot scale with sugar cane bagasse for bioethanol production Ind Crops Prod 2012 35 274 279 10.1016/j.indcrop.2011.07.010

[B58] Rodriguez-Zuniga UF Farinas CS Neto VB Couri S Crestana S *Aspergillus niger* production of cellulases by solid-state fermentation Pesqui Agropecu Bras 2011 46 912 919 10.1590/S0100-204X2011000800018

[B59] Kasana RC Salwan R Dhar H Dutt S Gulati A A rapid and easy method for the detection of microbial cellulases on agar plates using gram’s iodine Curr Microbiol 2008 57 503 507 10.1007/s00284-008-9276-8 18810533

[B60] Saqib AAN Whitney PJ Esculin gel diffusion assay (EGDA): A simple and sensitive method for screening β-glucosidases Enzyme Microb Technol 2006 39 182 184 10.1016/j.enzmictec.2005.09.013

[B61] Mandels M Reese ET Induction of cellulase in fungi by cellobiose J Bacteriology 2001 73 816 826 10.1128/jb.79.6.816-826.1960 PMC278786 14420566

[B62] Xiao Z Storms R Tsang A Microplate-based filter paper assay to measure total cellulase activity Biotechnol Bioeng 2004 88 832 837 10.1002/bit.20286 15459905

[B63] Miller GL Use of dinitrosalicylic acid reagent for determination of reducing sugar Anal Chem 1959 31 426 428 10.1021/ac60147a030

[B64] Barnett HC Hunter BB Illustrated genera of imperfect fungi 1999 New York: Macmillan Publishing Company

[B65] De Hoog GS Guarro J Gené J Figueras MJ Atlas of clinical fungi 2000 Centraalbureau voor Schimmelcultures, Utrecht: Universitat Rovira I Virgili, Reus

[B66] Larone DH Medically important fungi: a guide to identification 2002 Washington: ASM Press

[B67] Kern MA Blevins KS Micologia médica 1999 São Paulo: Premier

[B68] AHG G v d e de Hoog GS Variability and molecular diagnostics of the neurotropic species *Cladophialophora bantiana* Stud Mycol 1999 43 151 162

[B69] White TJ Bruns T Lee S Taylor J Innis MA, Gelfand DH, Sninsky JJ, White TJ PCR protocols: a guide to methods and applications Amplification and direct sequencing of fungal ribosomal RNA genes for phylogenetics 1990 New York: Academic Press 315 322

[B70] Glass NL Donaldson GC Development of primer sets designed for use with the PCR to amplify conserved genes from filamentous ascomycetes Appl Environ Microbiol 1995 61 1323 1330 7747954 10.1128/aem.61.4.1323-1330.1995 PMC167388

[B71] Staden R The Staden sequence analysis package Mol Biotechnol 1996 5 233 241 10.1007/BF02900361 8837029

[B72] Tamura K Dudley J Nei M Kumar S MEGA 4: molecular evolutionary genetics analysis Mol Biol Evol 2007 24 1596 1599 10.1093/molbev/msm092 17488738

[B73] Saitou N Nei M The neighbor-joining method: a new method for reconstructing phylogenetic trees Mol Biol Evol 1987 4 406 425 3447015 10.1093/oxfordjournals.molbev.a040454

[B74] Jukes TH Cantor CR Munro HN Evolution of protein molecules Mammalian protein metabolism 1969 New York: Academic Press 21 132

