# Supplementary material for: The capability of endophytic fungi for production of hemicellulases and related enzymes
Source: BMC Biotechnol. 2013 Oct 31;13:94. doi: 10.1186/1472-6750-13-94 (PMC3840621; doi:10.1186/1472-6750-13-94)
Supplement: Additional file 1: Table S1 — Hydrolysis rate of the bioprospected fungal strains. Table S2. Nucleotide sequences of fungal strains submitted to GenBank. [file 1472-6750-13-94-S1.docx]

**The capability of endophytic fungi for xylanase and accessory enzymes production**

Diogo Robl^a,b*^, Priscila da Silva Delabona^b^, Carla Montanari Mergel^a^, Juan Diego Rojas^a^, Patrícia dos Santos Costa^b^, Ida Chapaval Pimentel^c^, Vania Aparecida Vicente^c^, José Geraldo da Cruz Pradella^b^, Gabriel Padilla^a*^

**Table 1S**: Strains used in the phylogenic analysis. Nucleotide sequences were obtained/submitted to GenBank

| **Name** | **Reference** | **Genes sequenced/Genebank access** | **Source** | **Geography** |
| --- | --- | --- | --- | --- |
| *Aspergillu niger* | DR02 | ITS - KC311839, BT2 - KC311845 | *P. orientalis* | Brazil, Curitiba |
| *Annuhypoxylon stygium* | DR47 | ITS - KC311843, BT2 - KC311846 | *E. benthamii* | Brazil, Colombo |
| *Penicillium kloeckeri* | DR49 | ITS - KC311844, BT2 - KC311847 | Spoiled books | Brazil, Joinville |
| *Alternaria* sp. | DR40 | ITS - KC311842 | *E. benthamii* | Brazil, Colombo |
| *Trichoderma atroviride* | DR17 | ITS - KC311840 | *E. benthamii* | Brazil, Colombo |
| *Trichoderma atroviride* | DR19 | ITS - KC311841 | *E. benthamii* | Brazil, Colombo |
| *Aspergillus niger* | CBS 554.65 | ITS - AJ223852, BT2 - GU296687 | Tannin-gallic acid fermentation | USA, Connecticut |
| *Aspergillus niger* | CBS 120.49 | ITS - AJ280006, BT2 - GU296688 | Unkown | USA |
| *Aspergillus aculeatus* | CBS 172.66 | ITS - AJ279988, BT2 - FJ629271 | Tropical soil | Unknown |
| *Aspergillus japonicus* | CBS 114.51 | ITS - AJ279985, BT2 - GU296707 | Saito 5087 | Unknown |
| *Aspergillus tubingensis* | CBS 134.48 | ITS - AJ223853, BT2 - GU296696 | Unknown | Unknown |
| *Aspergillus tubingensis* | CBS 127.49 | ITS - AJ280007 | *Coffea arabica*, seed | Unknown |
| *Aspergillus tubingensis* | CBS 110.42 | BT2 - DQ768455 | Unknown | Unknown |
| *Aspergillus foetidus* | CBS 564.65 | ITS - AJ280009 , BT2 - GU296697 | Unknown | Japan |
| *Aspergillus brasiliensis* | IMI 381727 | ITS - AJ280010, BT2 - AM295186 | Soil | Brazil, São Paulo, Pedreira |
| *Aspergillus carbonarius* | NRRL 67 | ITS - U65305, BT2 - EF661097 | Unknown | Unknown |
| *Aspergillus heteromorphus* | CBS 117.55 | ITS - AJ280013, BT2 - GU296704 | Culture contaminant | Brazil |
| *Aspergillus ellipticus* | CBS 707.79 | ITS - AJ280014, BT2 - FJ629279 | Soil | Costa Rica |
| *Annulohypoxylon stygium* | E6826d | ITS - HQ008900 | *Macrocarpaea sodiroana* | Ecuador |
| *Annulohypoxylon stygium* | BCRC34024 | BT2 - AY951667 | Unknown | Tawain |
| *Annulohypoxylon stygium* | BCRC34023 | BT2 - AY951666 | Unknown | Tawain |
| *Annulohypoxylon stygium* var *annulatum* | BCRC34025 | BT2 - AY951669 | Unknown | France |
| *Annulohypoxylon urceolatum* | SUT098 | ITS - DQ322103 | Unknown | Thailand, Songkhla Province |
| *Annulohypoxylon urceolatum* | BCRC34028 | BT2 - AY951670 | Unknown | Tawain |
| *Annulohypoxylon nitens* | BCRC34021 | ITS - EF026138, BT2 - AY951663 | Unknown | Tawain, Taipei |
| *Annulohypoxylon bovei* var. *microspora* | BCRC34012 | ITS - EF026141, BT2 - AY951654 | Unknown | Tawain |
| *Annulohypoxylon squamulosum* | BCRC34022 | ITS - EF026139, BT2 - AY951665 | Unknown | Tawain |
| *Annulohypoxylon moriforme* var. *microdiscus* | BCRC34018 | ITS - EF026137, BT2 - AY951660 | Unknown | Tawain |
| *Annulohypoxylon multiforme* | ATCC 36665 | ITS - AF201717 | *Betula* sp. | - |
| *Annulohypoxylon cohaerens* | 3041 | ITS - EF026140 | On *Fagus* sp. | France, Ariège, Rimont |
| *Annulohypoxylon elevatidiscus* | BCRC34014 | BT2 - AY951656 | Unknown | Tawain |
| *Hypoxylon investiens* | CBS 118185 | ITS - FJ185308, BT2 - FJ185299 | Decorticated and blackened branch | Ecuador |
| *Talaromyces wortmannii* | KUC1286 | ITS - HM469393 | Wood | Korea |
| *Talaromyces wortmannii* | CBS 391.48 | ITS - JN899352 | Unknown | Unknown |
| *Talaromyces wortmannii* | W35 | BT2 - AY533533 | Unknown | Unknown |
| *Talaromyces radicus* | CBS 100489 | ITS - JN899324 | Root of seedling of *Triticum aestivum* | Australia, Wagga |
| *Talaromyces allahabadensis* | CBS 453.93 | ITS - JN899345 | Soil of cultivated field | India, Allahabad |
| *Talaromyces tardifaciens* | CBS 250.94 | ITS - JN899361 | Unknown | - |
| *Talaromyces loliensis* | CBS 643.80 | ITS - JN899379 | *Lolium* sp. | New Zealand, Palmerston North |
| *Talaromyces phialosporus* | CBS 233.60 | ITS - JN899340, BT2 - HQ156949 | Milled Californian rice | USA, California |
| *Talaromyces variabilis* | CBS 385.48 | ITS - JN899343 | Cocos fibre | South Africa, Johannesburg |
| *Talaromyces islandicus* | CBS 338.48 | ITS - JN899318 | Unknown source | South Africa, Cape Town |
| *Talaromyces rugulosus* | CBS 371.48 | ITS - JN89937 | *Solanum tuberosum* | USA, , Connecticut |
| *Talaromyces amestolkiae* | CBS 884.72 | BT2 - JX315622 | Manure | France, |
| *Talaromyces ruber* | CBS 113138 | BT2 - JX965349 | PVC/Paper wall covering | - |
| *Talaromyces bacillisporus* | CBS 296.48 | BT2 - AY753368 | *Begonia* sp., leaf | USA, New York city |
| *Talaromyces palmae* | CBS 442.88 | BT2 - HQ156947 | *Begonia* sp., leaf | USA, New York city |
| *Talaromyces minioluteus* | CV0383 | BT2 - JF910277 | Sandy fynbos soil | South Africa, Western Cape |
| *Talaromyces purpurogenus* | CBS 286.36 | BT2 - JX315639 | Parasitic on a culture of *Aspergillus oryzae* | Japan |
| *Talaromyces purpurogenus* | CBS 184.27 | BT2 - JX315637 | Soil | USA, Louisiana |
| *Talaromyces stollii* | CBS 132706 | BT2 - JX965359 | Indoor air form bakery | The Netherlands, Avenhorn |
| *Talaromyces pinophilus* | L14 | BT2 - EU597716 | Litchi | South Africa |
| *Alternaria alternata* | CBS 112018 | ITS - AY673074 | Phaeohyphomycosis | Spain, Santiago de Compostela |
| *Alternaria porri* | ATCC 58175 | ITS - AF229470 | *Allium fistulosum* | USA, Arizona |
| *Alternaria tenuissima* | ATCC 16423 | ITS - AF229476 | Unkown | Unkown |
| *Alternaria longipes* | EGS 30-033 | ITS - AY278835 | Unkown | Unkown |
| *Alternaria arborescens* | EGS 39-128 | ITS - AF347033 | Unkown | Unkown |
| *Alternaria destruens* | EGS 46-069 | ITS - AY278836 | Unkown | Unkown |
| *Alternaria solani* | CBS 111.44 | ITS - Y17070 | *Ageratum houstonianum*, seed | Unkown |
| *Alternaria brassicicola* | CBS 125088 | ITS - GQ496082 | *Brassica oleracea*, leaf | Hungary,Keszthely |
| *Alternaria dauci* | CCRC33651 | ITS - AF267130 | Seed | Unkown |
| *Alternaria crassa* | DGG Acr1 | ITS - AF229464 | Unkown | Unkown |
| *Alternaria japonica* | ATCC 13618 | ITS - AY376639 | Infected radish | Canada |
| *Trichoderma atroviride* | CBS 142.95 | ITS - AF456917 | Gallery of ambrosia beetle, in decayed log of *Quercus* sp. | Slovenia |
| *Trichoderma atroviride* | DAOM 179514 | ITS - EU280125 | Unkown | Unkown |
| *Hypocrea lixii* | CBS 226.95 | ITS - AF057606 | Soil | England |
| *Hypocrea viridescens* | CBS 433.34 | ITS - AY380905 | mouldy apple core | UK |
| *Trichoderma aggressivum* | CBS 689.94 | ITS - FJ442606 | Mushroom compost | England |
| *Trichoderma longibrachiatum* | CBS 816.68 | ITS - EU401556 | Mud | USA, Ohio |
| *Hypocrea koningii* | CBS 979.70 | ITS - DQ323410 | Decaying angiosperm wood | The Netherlands, Groeneveld |
| *Hypocrea vinosa* | CBS 960.68 | ITS - AF191038 | Sand, in lysimeter system | USA, Ohio, Cincinnati |
| *Trichoderma hamatum* | ATCC 28012 | ITS - X93975 | Soil | USA, North Carolina |
| *Trichoderma virens* | ATCC MYA-4894 | ITS - JX174053 | Unkown | Unkown |

**Table 2S.**  Results of the selection of strains using the sum of the hydrolysis ratios for liquor agar and xylan agar, and calculation of the average halos obtained in the esculin gel diffusion assay (EGDA)

| **Strain** | **Identification^a^** | **Source** | **Hydrolysis ratio using liquor agar^b,c^** | **Hydrolysis ratio using xylan agar^b,c^** | **Ratio sum^b,c^** | **EGDA halo average (mm)^d,e^** |
| --- | --- | --- | --- | --- | --- | --- |
| ATCC64973 | *A. niger* | *-* | 1.00 | 1.42 | 2.42 | 14.50 |
| DR01 | *Aspergillus* sp. | Spoiled books | 0.00 | 1.82 | 1.82 | 16.00 |
| DR02 | *Aspergillus niger* | *P. orientalis* | 2.48 | 1.59 | 4.06 | 17.25 |
| DR03 | *Aspergillus* sp. | *E. benthamii* | 2.44 | 1.00 | 3.44 | - |
| DR04 | *Paecilomyces lilacinus* | *G. max* | 0.00 | 1.85 | 1.85 | 13.00 |
| DR05 | *Trichoderma* sp. | *E. benthamii* | 1.00 | 1.83 | 2.83 | + |
| DR06 | *Aspergillus* sp. | Spoiled books | 2.47 | 1.57 | 4.04 | 11.50 |
| DR07 | NI | *E. benthamii* | 2.89 | 1.00 | 3.89 | 17.25 |
| DR08 | *Aspergillus* sp. | *E. benthamii* | 2.32 | 2.13 | 4.45 | + |
| DR09 | NI | *E. benthamii* | 1.86 | 1.61 | 3.47 | 19.00 |
| DR100 | NI | *S. tuberosum* | 0.00 | 1.38 | 1.38 | + |
| DR101 | NI | *E. benthamii* | 0.00 | 1.36 | 1.36 | - |
| DR102 | NI | *E. benthamii* | 0.00 | 1.36 | 1.36 | - |
| DR103 | *Cladosporium* sp. | *G. max* | 0.00 | 1.35 | 1.35 | + |
| DR104 | *Penicillium* sp. | *G. max* | 0.00 | 1.35 | 1.35 | - |
| DR105 | *Penicillium janthinellum* | *S. officinarum* | 0.00 | 1.33 | 1.33 | 14.25 |
| DR106 | *Cladosporium* sp. | *G. max* | 0.00 | 1.32 | 1.32 | - |
| DR107 | *Penicillium* sp. | *G. max* | 0.00 | 1.32 | 1.32 | - |
| DR108 | *Aspergillus* sp. | *G. max* | 0.00 | 1.31 | 1.31 | 12.00 |
| DR109 | *Penicillium* sp. | *G. max* | 0.00 | 1.29 | 1.29 | 12.00 |
| DR110 | NI | *E. benthamii* | 0.00 | 1.27 | 1.27 | 13.50 |
| DR111 | *Trichoderma* sp. | *E. benthamii* | 0.00 | 1.21 | 1.21 | - |
| DR112 | *Saccharicola* sp. | *S. officinarum* | 0.00 | 1.20 | 1.20 | - |
| DR113 | *Alternaria* sp. | *P. orientalis* | 0.00 | 1.14 | 1.14 | + |
| DR114 | NI | *E. benthamii* | 0.00 | 1.12 | 1.12 | 12.25 |
| DR115 | *Bipolaris* sp. | *G. max* | 0.00 | 1.11 | 1.11 | + |
| DR116 | *Rhizomucor* sp. | *G. max* | 0.00 | 1.00 | 1.00 | - |
| DR117 | NI | *E. benthamii* | 0.00 | 1.00 | 1.00 | - |
| DR118 | NI | *E. benthamii* | 0.00 | 1.00 | 1.00 | 12.50 |
| DR119 | *Beauveria bassiana* | *G. max* | 0.00 | 1.00 | 1.00 | + |
| DR12 | *Penicillium* sp. | *E. benthamii* | 2.82 | 1.55 | 4.36 | + |
| DR120 | *Trichoderma* sp. | *S. officinarum* | 0.00 | 1.00 | 1.00 | + |
| DR121 | *Diaphorte* sp. | *S. officinarum* | 0.00 | 0.00 | 0.00 | - |
| DR13 | *Penicillium* sp. | *G. max* | 0.00 | 1.00 | 1.00 | 14.00 |
| DR14 | *Aspergillus* sp. | *G. max* | 0.00 | 1.00 | 1.00 | - |
| DR15 | *Fusarium* sp. | *G. max* | 1.58 | 1.40 | 2.98 | - |
| DR16 | NI | *E. benthamii* | 1.23 | 1.54 | 2.77 | - |
| DR17 | *T. atroviride* | *E. benthamii* | 1.73 | 1.05 | 2.78 | + |
| DR18 | *Penicillium* sp. | *G. max* | 1.17 | 1.82 | 2.99 | - |
| DR19 | *T. atroviride* | *E. benthamii* | 2.00 | 1.00 | 3.00 | 12.00 |
| DR20 | *Coletotrichum gloeosporioides* | *G. max* | 0.00 | 1.00 | 1.00 | 20.00 |
| DR21 | NI | *E. benthamii* | 2.00 | 1.59 | 3.59 | 20.00 |
| DR22 | *Acremonium* sp. | Spoiled books | 1.50 | 1.16 | 2.66 | 12.00 |
| DR23 | *C. gloeosporioides* | *G. max* | 1.83 | 1.38 | 3.21 | 17.50 |
| DR24 | *Aspergillus* sp. | *G. max* | 1.65 | 1.42 | 3.07 | 18.00 |
| DR25 | *Aspergillus* sp. | *E. benthamii* | 1.92 | 1.38 | 3.31 | 13.25 |
| DR26 | *Paecilomyces sp.* | *G. max* | 2.33 | 1.45 | 3.79 | 15.00 |
| DR27 | *Penicillium* sp. | *E. benthamii* | 2.29 | 1.42 | 3.71 | + |
| DR28 | *Penicillium* sp. | *G. max* | 0.00 | 3.78 | 3.78 | - |
| DR29 | *Aspergillus* sp. | *G. max* | 1.32 | 1.84 | 3.16 | - |
| DR30 | *Aspergillus* sp. | *E. benthamii* | 1.65 | 1.60 | 3.25 | 14.50 |
| DR31 | *Aspergillus* sp. | *G. max* | 2.29 | 1.24 | 3.53 | - |
| DR32 | NI | *E. benthamii* | 0.00 | 1.00 | 1.00 | - |
| DR33 | *Aspergillus* sp. | *G. max* | 2.22 | 1.24 | 3.46 | 16.00 |
| DR34 | *Aspergillus* sp. | *G. max* | 1.00 | 2.75 | 3.75 | 12.50 |
| DR35 | *Aspergillus* sp. | *G. max* | 1.00 | 2.75 | 3.75 | 18.25 |
| DR36 | *Aspergillus* sp. | *G. max* | 2.24 | 1.40 | 3.64 | 16.00 |
| DR37 | *Trichoderma* sp. | *G. max* | 1.39 | 1.24 | 2.64 | 12.75 |
| DR38 | *Trichoderma* sp. | *E. benthamii* | 1.37 | 1.34 | 2.71 | 11.00 |
| DR39 | *Acremonium* sp. | *G. max* | 1.25 | 1.28 | 2.53 | 15.00 |
| DR40 | *Alternaria* sp. | *E. benthamii* | 0.00 | 1.28 | 1.28 | 12.00 |
| DR41 | NI | *S. officinarum* | 0.00 | 1.55 | 1.55 | 16.00 |
| DR42 | *Alternaria* sp. | *E. benthamii* | 1.58 | 1.08 | 2.66 | 13.00 |
| DR43 | NI | *E. benthamii* | 0.00 | 1.00 | 1.00 | 16.00 |
| DR44 | *Aspergillus* sp. | *G. max* | 0.00 | 0.00 | 0.00 | - |
| DR45 | *Alternaria* sp. | *E. benthamii* | 0.00 | 1.22 | 1.22 | 14.50 |
| DR46 | NI | *E. benthamii* | 0.00 | 1.58 | 1.58 | 18.50 |
| DR47 | *A. stygium* | *E. benthamii* | 1.00 | 1.72 | 2.72 | 20.50 |
| DR48 | NI | *E. benthamii* | 0.00 | 1.45 | 1.45 | 17.00 |
| DR49 | *P. kloeckeri* | Spoiled books | 0.00 | 1.53 | 1.53 | 15.00 |
| DR50 | *Penicillium* sp. | *G. max* | 1.51 | 1.19 | 2.70 | - |
| DR51 | *Aspergillus* sp. | *E. benthamii* | 1.27 | 1.33 | 2.60 | + |
| DR52 | *Penicillium* sp. | *G. max* | 0.00 | 1.43 | 1.43 | 15.00 |
| DR53 | *Penicillium* sp. | *G. max* | 0.00 | 1.36 | 1.36 | 14.50 |
| DR54 | *Aspergillus* sp. | *G. max* | 1.56 | 1.25 | 2.80 | + |
| DR55 | *Aspergillus* sp. | *G. max* | 0.00 | 1.09 | 1.09 | 16.50 |
| DR56 | *Aspergillus* sp. | *G. max* | 0.00 | 1.35 | 1.35 | 17.00 |
| DR57 | *Aspergillus* sp. | *G. max* | 0.00 | 1.34 | 1.34 | 17.00 |
| DR58 | *Aspergillus* sp. | *G. max* | 1.00 | 1.67 | 2.67 | 18.50 |
| DR59 | NI | *E. benthamii* | 1.00 | 1.63 | 2.63 | + |
| DR60 | *Penicillium* sp. | *G. max* | 1.24 | 1.31 | 2.55 | 12.00 |
| DR61 | NI | *E. benthamii* | 1.00 | 1.49 | 2.49 | 20.00 |
| DR62 | *Penicillium* sp. | *G. max* | 1.00 | 1.47 | 2.47 | 12.50 |
| DR63 | *Coletotrichum* sp. | *G. max* | 1.13 | 1.33 | 2.47 | - |
| DR64 | *Penicillium* sp. | *G. max* | 1.00 | 1.46 | 2.46 | + |
| DR65 | *Penicillium* sp. | *G. max* | 1.00 | 1.46 | 2.46 | - |
| DR66 | NI | *E. benthamii* | 1.23 | 1.17 | 2.40 | + |
| DR67 | NI | *S. officinarum* | 0.00 | 2.35 | 2.35 | 13.50 |
| DR68 | *Penicillium* sp. | *G. max* | 1.37 | 0.96 | 2.33 | 11.00 |
| DR69 | NI | *E. benthamii* | 0.00 | 2.00 | 2.00 | - |
| DR70 | *Penicillium* sp. | *G. max* | 0.00 | 2.00 | 2.00 | + |
| DR71 | NI | *E. benthamii* | 1.00 | 1.00 | 2.00 | - |
| DR72 | *Aspergillus* sp. | Spoiled books | 0.00 | 1.94 | 1.94 | + |
| DR73 | NI | *G. max* | 0.00 | 1.87 | 1.87 | - |
| DR74 | NI | *S. officinarum* | 0.00 | 1.86 | 1.86 | - |
| DR75 | *Cladosporium* sp. | *G. max* | 0.00 | 1.74 | 1.74 | - |
| DR76 | *Penicillium* sp. | *G. max* | 0.00 | 1.73 | 1.73 | + |
| DR77 | *Alternaria* sp. | *E. benthamii* | 0.00 | 1.73 | 1.73 | 16.00 |
| DR78 | *Cladosporium* sp. | *G. max* | 0.00 | 1.70 | 1.70 | - |
| DR79 | NI | *E. benthamii* | 0.00 | 1.67 | 1.67 | + |
| DR80 | *P. lilacinus* | *S. tuberosum* | 0.00 | 1.67 | 1.67 | + |
| DR81 | *P. janthinellum* | *S. officinarum* | 0.00 | 1.58 | 1.58 | - |
| DR82 | NI | *E. benthamii* | 0.00 | 1.58 | 1.58 | - |
| DR83 | NI | Spoiled books | 0.00 | 1.57 | 1.57 | + |
| DR84 | *Cladosporium* sp. | Spoiled books | 0.00 | 1.55 | 1.55 | - |
| DR85 | *Phomopsis* sp. | *S. officinarum* | 0.00 | 1.55 | 1.55 | + |
| DR86 | NI | *E. benthamii* | 0.00 | 1.54 | 1.54 | - |
| DR87 | *P. lilacinus* | *G. max* | 0.00 | 1.54 | 1.54 | + |
| DR88 | *Penicillium* sp. | *G. max* | 0.00 | 1.51 | 1.51 | - |
| DR89 | *Cladosporium* sp. | *G. max* | 0.00 | 1.48 | 1.48 | - |
| DR90 | *Chaetomium* sp. | *G. max* | 0.00 | 1.47 | 1.47 | - |
| DR91 | *Penicillium* sp. | *G. max* | 0.00 | 1.43 | 1.43 | 12.00 |
| DR92 | *Cladosporium* sp. | *G. max* | 0.00 | 1.43 | 1.43 | - |
| DR93 | NI | Spoiled books | 0.00 | 1.43 | 1.43 | + |
| DR94 | *Aspergillus* sp. | *G. max* | 0.00 | 1.43 | 1.43 | - |
| DR95 | NI | *G. max* | 0.00 | 1.42 | 1.42 | - |
| DR96 | *Alternaria* sp. | *E. benthamii* | 0.00 | 1.41 | 1.41 | 17.50 |
| DR97 | NI | Spoiled books | 0.00 | 1.41 | 1.41 | 11.50 |
| DR98 | *Fusarium* sp. | *S. officinarum* | 0.00 | 1.41 | 1.41 | - |
| DR99 | *Fusarium* sp. | *S. officinarum* | 0.00 | 1.39 | 1.39 | 17.75 |

^a^NI = Not identified; ^b^0.00 = No growth; ^c^1.00 = Growth and absence of hydrolysis halo; ^d^+ = Positive unmeasured halo; ^e^- = No halo.
